# Reconstitution of Intestinal CD4 and Th17 T Cells in Antiretroviral Therapy Suppressed HIV-Infected Subjects: Implication for Residual Immune Activation from the Results of a Clinical Trial

**DOI:** 10.1371/journal.pone.0109791

**Published:** 2014-10-23

**Authors:** Gabriella d'Ettorre, Silvia Baroncelli, Luca Micci, Giancarlo Ceccarelli, Mauro Andreotti, Prachi Sharma, Gianfranco Fanello, Fausto Fiocca, Eugenio Nelson Cavallari, Noemi Giustini, Alessandra Mallano, Clementina M. Galluzzo, Stefano Vella, Claudio M. Mastroianni, Guido Silvestri, Mirko Paiardini, Vincenzo Vullo

**Affiliations:** 1 Department of Public Health and Infectious Diseases, Istituto Pasteur – Fondazione Cenci-Bolognetti, University of Rome “Sapienza”, Rome, Italy; 2 Department of Therapeutic Research and Medicines Evaluation, Istituto Superiore di Sanità, Rome, Italy; 3 Division of Microbiology and Immunology, Yerkes National Primate Research Center, Atlanta, Georgia, United States of America; 4 Division of Pathology, Yerkes National Primate Research Center, Atlanta, Georgia, United States of America; 5 Department of Emergency Surgery- Emergency Endoscopic Unit, Policlinico Umberto I, University of Rome “Sapienza”, Rome, Italy; 6 Infectious Disease Unit, Fondazione Eleonora Lorillard Spencer Cenci, Sapienza University, Latina, Italy; 7 Department of Pathology and Laboratory Medicine, Emory University School of Medicine, Atlanta, Georgia, United States of America; University of Rome Tor Vergata, Italy

## Abstract

**Introduction:**

During HIV infection the severe depletion of intestinal CD4**^+^** T-cells is associated with microbial translocation, systemic immune activation, and disease progression. This study examined intestinal and peripheral CD4**^+^** T-cell subsets reconstitution under combined antiretroviral therapy (cART), and systemic immune activation markers.

**Methods:**

This longitudinal single-arm pilot study evaluates CD4^+^ T cells, including Th1 and Th17, in gut and blood and soluble markers for inflammation in HIV-infected individuals before (M0) and after eight (M8) months of cART. From January 2010 to December 2011, 10 HIV-1 naïve patients were screened and 9 enrolled. Blood and gut CD4^+^ T-cells subsets and cellular immune activation were determined by flow-cytometry and plasma soluble CD14 by ELISA. CD4^+^ Th17 cells were detected in gut biopsies by immunohistochemistry. Microbial translocation was measured by limulus-amebocyte-lysate assay to detect bacterial lipopolysaccharide (LPS) and PCR Real Time to detect plasma bacterial 16S rDNA.

**Results:**

Eight months of cART increased intestinal CD4**^+^** and Th17 cells and reduced levels of T-cell activation and proliferation. The magnitude of intestinal CD4**^+^** T-cell reconstitution correlated with the reduction of plasma LPS. Importantly, the magnitude of Th17 cells reconstitution correlated directly with blood CD4**^+^** T-cell recovery.

**Conclusion:**

Short-term antiretroviral therapy resulted in a significant increase in the levels of total and Th17 CD4**^+^** T-cells in the gut mucosa and in decline of T-cell activation. The observation that pre-treatment levels of CD4**^+^** and of CD8**^+^** T-cell activation are predictors of the magnitude of Th17 cell reconstitution following cART provides further rationale for an early initiation of cART in HIV-infected individuals.

**Trial Registration:**

ClinicalTrials.gov NCT02097381

## Introduction

HIV infection is characterized by a progressive depletion of CD4**^+^** T cells, a severe dysregulation of the immune system function and progression to AIDS. When available, the modern cART has transformed HIV infection in a manageable chronic disease. Nevertheless, HIV individuals with access to cART regimens continue to have a 10-years shorter life expectancy [1], [2], and appear to be more prone to cardiovascular, liver, and renal diseases [3], than people without HIV. This higher morbidity and mortality has been associated to a status of immune activation/inflammation that persist despite effective inhibition of viral replication achieved by cART [4]. Indeed, persistent immune system activation/inflammation and higher levels of microbial translocation associate with a poor recovery of CD4**^+^** T cells in individuals cART-suppressed for many years [5]–[9]. The causes of persistent systemic inflammation are under extensive investigation, with a large number of studies focalizing on the possible role of mucosal immune dysfunction and of depletion of intestinal CD4**^+^** T cells [10]–[13]. A specific subset of CD4**^+^** T cells, named Th17, is specialized to maintain mucosal integrity and to produce a robust antimicrobial inflammatory response [14]. Th17 cells constitute a distinct lineage from Th1 and Th2 and are characterized by the production of signature cytokines – IL-17A, IL-17F, IL-22 - and the expression of the transcription factor RORgt [15]–[21]. Th17 cells stimulate neutrophil recruitment, proliferation of epithelial cells, production of tight junction proteins and antimicrobial defensins [22]–[24].

Cross sectional studies clearly showed that intestinal Th17 cells are severely depleted in chronically HIV infected subjects, with the severity of Th17 cell loss being associated with the extents of immune activation, microbial translocation, and disease progression [12], [25]–[29]. Consistent with the pathogenic role of intestinal Th17 cell loss are the findings generated in the nonhuman primate models of HIV infection. Indeed, in the pathogenic SIV infection of macaques a preferential depletion of intestinal Th17 cells has been associated with immune activation, dissemination of bacterial products from the intestine to the systemic circulation, and progression to AIDS [30]–[32]. Moreover, and in contrast to what found in HIV-infected humans and SIV-infected macaques, intestinal Th17 cells are preserved at healthy frequencies in SIV-infected sooty mangabeys, African monkey species natural hosts for the virus that preserve mucosal integrity, avoid chronic immune activation and do not progress to AIDS despite high levels of viral replication [18], [25], [33]–[35]. Recently, preservation of intestinal Th17 cells has been shown also in HIV-infected patients who are able to spontaneously control HIV replication without cART (so called “Elite controllers” and long term non progressors”) [28], [29], [36]–[38]. Finally, in rhesus macaques increased size of the Th17 compartment prior to SIV infection associated with reduced levels of SIV replication and increased mucosal integrity in the first weeks of infection [39]. Collectively, these studies highlight the importance of preserving mucosal integrity and Th17 cells during HIV infection. Thus, it is very relevant to understand the efficacy of cART to reconstitute intestinal CD4**^+^** T cells in HIV-infected individuals. Unfortunately, since the gastrointestinal tissues are not easily accessible for analysis, data on cART-induced reconstitution of human intestinal CD4**^+^** T cells in general, and of Th17 in particular, are still largely incomplete. In this longitudinal non randomized pilot study we evaluated the reconstitution of total, Th1 and Th17 CD4**^+^** T-cells colon samples and in blood collected in nine HIV-infected patients before (M0) and at eight months of cART (M8). Furthermore, we investigated the association between the extent of the repopulation of these T cell subsets and the extent of systemic immune activation.

## Methods

### Study design, recruitment and study eligibility criteria

**Figure 1:**
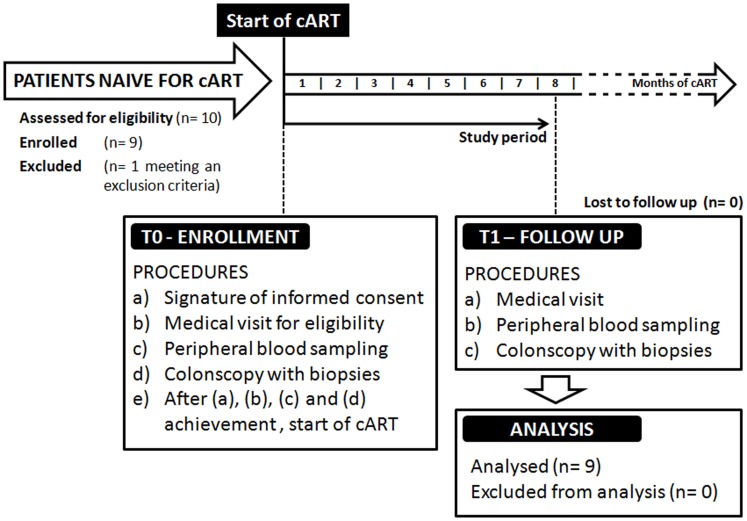
The flowchart of the clinical trial. Experimental design of the study.

The protocol for this trial and supporting CONSORT checklist are available as supporting information; see Checklist S1, Protocol S1 and S2. This is a longitudinal, single arm, pilot study (Figure 1). From January 2010 to December 2011, ten HIV positive patients were recruited in a single clinical site, the Division of Infectious Diseases, Department of Public Health and Infectious Diseases, Hospital of the University “Sapienza” of Rome (Italy).

The inclusion criteria allowed to enroll HIV-positive patients i) who have signed the informed consent, ii) aged between 18 and 60 years, iii) naïve for antiretroviral treatment who met the criteria to start cART according to International Guidelines. Exclusion criteria were: i) treatment with glucocorticosteroids and any immune modulating medication for more than seven days in the previous month, ii) any past or current systemic malignancy, iii) history of or current inflammatory diseases of the small or large intestine, iv) anemia, v) diarrhea, vi) use of anticoagulants, vii) pregnancy and viii) any contraindications to phlebotomy or colonscopy.

This study is registered on the ClinicalTrials.gov registry with identifier number NCT02097381. The approval of the independent Ethics Committee is the only form of registration required by Italian law; for this reason, the registration in an international registry was made after patient recruitment.

Moreover the authors confirm that all ongoing and related trials for this intervention are registered.

### Ethics Statement

The study was approved by the institutional review board (Department of Public Health and Infectious Diseases, University of Rome “Sapienza” and Ethics Committee Azienda Policlinico Umberto I of Rome). All study participants gave informed written informed consent.

### Laboratory procedures, data collection and analysis

#### Sampling

Patients were sampled for peripheral blood (20 ml) and underwent endoscopic procedures before the start of cART (M0) and after eight months of treatment (M8). Colonic washing was carried out by PEG administration 24 hours before the examination. Endoscopic procedure was performed with conscious sedation (midazolam 5 mg/i.v.) using large cup forceps (Radial Jaw 4, Boston Scientific, Natick, Massachusetts, USA). All patients underwent a total colonoscopy and retrograde ileoscopy for at least 10 cm of distal ileum with conventional or slim scope (model CF or PCF-160 AI, Olympus Medical Europe GmbH, Hamburg, Germany). We obtained specimens (6 biopsies each site) from terminal ileum, ascending, descending and sigmoid colon.

#### Specimen Processing

Blood was processed the same day of phlebotomy to obtain PBMC by Ficoll gradient centrifugation (Lympholyte, Cederline Laboratories, Hornby, Ontario, Canada). For the immunological studies biopsies were pooled from each gut site and processed the same day of colonoscopy. Briefly, biopsies collected in RPMI 1640 were washed twice with EDTA wash media and re-suspended in EDTA solution 5 mM. Supernatant containing intra-epitelial lymphocytes (IELs) were removed, and biopsies were digested by 1 hour incubation at 37°C with 1 mg/mL Collagenase solution (Sigma-Aldrich, Sigma Aldrich, Milan, Italy) and 1.5 U DNAse (Invitrogen, Carlsbad, CA), allowing the isolation of lamina propria lymphocytes (LPL) that were filtered through a 70 µm cell stainer. Cells obtained were counted and re-suspended.

#### Cell cultures

Isolated mononuclear cells from blood and gut biopsies were seeded at the concentration of 2×10^6^ cells/ml with RPMI media+10% heat inactivated fetal bovine serum (FBS) and cultured overnight at 37°C and 5% CO_2_ in presence of medium alone; phorbol myristyl acetate (PMA) (3 ng/ml Sigma Aldrich, Milan Italy) and ionomycin (1 µM Sigma Aldrich, Milan, Italy); Streptokinase (SK, 10 µg/ml, Sigma Aldrich Milan, Italy); or Candida albicans mannoprotein antigen (*Ca*, 10 µl/ml kindly provided by Dr A. Cassone, Istituto Superiore di Sanità, Rome, Italy). Brefeldine A (BFA, Sigma Aldrich, Milan, Italy) was added to all culture conditions. Cells were collected, washed and stained for T cell phenotype.

#### Monoclonal antibody and T cell phenotyping

Multiparameter flow cytoflorimetric analysis were performed on stimulated PBMC and LPL. Cells were fixed-permeabilized (Fixation Buffer and Permeabilization Wash Buffer, BioLegend Cambridge, UK) and stained with combinations of fluorocrome-labelled monoclonal antibodies: CD3-FITC, CD4-PE, IFN-gamma-PerCP and IL-17A-alexa-fluor-647 (BioLegend, Cambridge, UK). CD4^+^ Th1 and CD4^+^ Th17 cells were identified as CD3^+^CD4^+^ cells that express IFN-γ or IL-17A, respectively. Activated and/or proliferating cells were identified by staining for CD3, CD4, CD8, CD38, Ki67 and HLADR. Acquisitions was performed on a FACScalibur flow cytometer with Cell Quest software (Becton Dickinson, San Jose, CA, USA) and data were analyzed using FlowJo (Tree Star Inc.).

#### Measurement of plasma soluble biomarkers

Plasma samples were analyzed by Limulus Amebocyte Assay for LPS levels (Lonza Group, Switzerland) and by ELISA for soluble CD14 (sCD14, R&D Systems, Minneapolis, Minnesota, USA). Tests were performed according to the manufacturers' instructions. Quantitative polymerase chain reaction (PCR) was used to measure the levels of plasma bacterial 16S rDNA. A 20-µL amplification reaction consisted of TaqMan Fast Universal PCR Master Mix, 0.5 µmol/L forward and reverse primers, 0.32 µmol/L probe (338P: 5′-FAM-GCT GCC TCC CGT AGG AGT-BHQ1-3′). Degenerate forward (8F: 5′-AGT TTG ATC CTG GCT CAG-3′) and reverse (515R: 5′-GWA TTA CCG CGG CKG CTG-3′) primers were used to amplify DNA templates encoding 16S rRNA [40]. The DNA was amplified in triplicate, and mean values were calculated. A standard curve was created from serial dilutions of plasmid DNA containing known copy numbers of the template. The reaction conditions for amplification of DNA were 95°C for 5 min, followed by 45 cycles at 95°C for 15 s and at 60°C for 1 min. Intra-assay variability typically is <5%

#### Virological analysis

Plasma samples were analyzed for HIV-1 RNA copy number by VERSANT HIV-1 RNA 1.0 kPCR assay (Siemens), with a detection limit of 37 copies/ml.

#### Immunohistochemistry (IHC) for CD4 and IL-17

The formalin-fixed paraffin-embedded colon biopsies were sectioned at 4 µM thickness and slides prepared. After deparaffinization, the slides were rehydrated and antigen retrieval done by microwave treatment after quenching of endogenous peroxydases. The sections were incubated with a 1∶200 dilution of anti-CD4 rabbit monoclonal antibody (Epitomics Inc., Burlingame, CA) or 1∶100 dilution of anti-IL-17 polyclonal goat antibody (R&D Systems), followed by 1∶250 dilution of biotinylated anti-rabbit (Vector laboratories, Inc., Burlingame, CA) or anti-goat antibodies (Vector laboratories, Inc., Burlingame, CA) respectively. The reactions were detected by the development of a chromogenic substrate (Vulcan Fast Red, Biocare Medical) and the slides were counterstained with hematoxylin. These procedures were performed with relevant negative and positive controls. Slides were semiquantitatively analyzed by using an Olympus BX-41 light microscope and counting the number of positive cells (200× magnification) in five fields per slide. Slides were scored as “−” (less than 3 positive cells); “+” (3–10 positive cells); “++” (20–40 positive cells) and “+++” (more than 50 positive cells).

#### Statistical analysis

Statistical analyses and graphical presentation were done using SPSS software, version 20.00 (IBM, Somers, NY, USA). Longitudinal comparison between pre-cART and post-cART values was performed by Wilcoxon matched pair test. Results are given as medians, ranges and percentages. Linear regression with Spearman's correlation coefficient was used to evaluate correlations between quantitative variables. Differences were considered statistically significant when p≤0.05.

## Results

### Population characteristic

10 HIV-1 naïve patients were screened and 9 enrolled. One patient was not eligible because met an exclusion criteria (clinical picture of proctosigmoiditis in the colonscopy performed at enrolment). All 9 patients enrolled were white males, with median age of 41 years (range 27–63) and a median time of two years from HIV infection diagnosis. All subjects were naïve for antiretroviral therapy, negative for hepatitis B and/or C co-infections and other concomitant diseases. The characteristics of patients at baseline, including anagraphic data, transmission routes and stages of CDC classification are reported in Table 1. At enrollment all patients had detectable viremia (median 4.68 HIV-RNA log10 copies/mL, range 3.60–5.83) with wild–type genotype and a median CD4**^+^** T cell count of 367 cell/µl (range 145–720). Median nadir of CD4**^+^** T cell was 354 (range 145–519) and median zenith of 4.58 HIV-RNA log10 copies/mL (range 3.69–5.83).

**Table 1:** Characteristics of patient population.

Patient	Sex	Age	Transmission route	CDC
**1**	M	41	omosexual	A1
**2**	M	39	omosexual	A2
**3**	M	54	eterosexual	A2
**4**	M	27	omosexual	A2
**5**	M	32	eterosexual	A2
**6**	M	53	eterosexual	A2
**7**	M	49	bisexual	A2
**8**	M	63	bisexual	A3
**9**	M	40	eterosexual	A2

The characteristics of patient population at baseline including anagraphic data, transmission routes and CDC stages.

The levels of CD4**^+^** T cells, express as percentages of total CD3**^+^** T cells, were significantly lower (p = 0.009) in GI tract (median 10.1%; range 4.0–16.1) as compared to blood (median 16%; range 5.0–30.0). Of note, the percentages of intestinal (r = −0.667, p = 0.050) and circulating (r = −0.833, p = 0.008) CD4**^+^** T cells were inversely associated with plasma viral load (data not shown).

Antiretroviral therapy started after baseline sampling, and consisted in a tenofovir-emtricitabine NRTI backbone (TDF/FTC, 300/200 mg/ml, once a day) plus boosted protease inhibitor, lopinavir/ritonavir (LPV/r, 400/100 mg twice a day) or darunavir/ritonavir (DRV/r 800/100 mg once a day).

### Impact of short-term cART on plasma viral load and CD4 T cell levels

**Figure 2:**
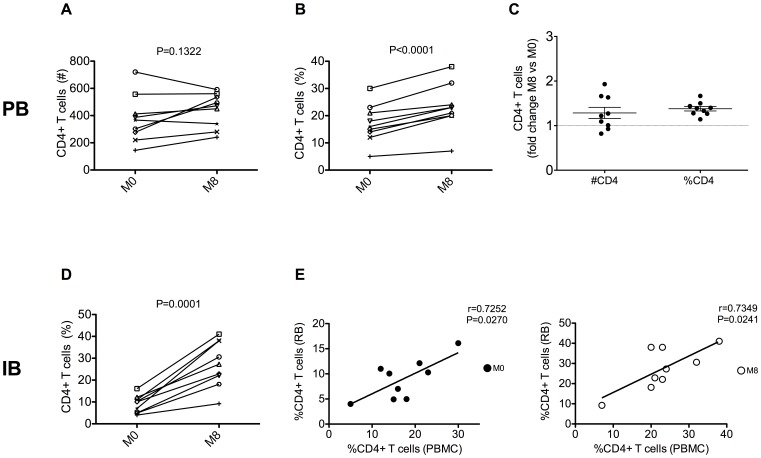
Effects of short-term cART on peripheral and intestinal CD4 T cell levels. Longitudinal assessment of CD4 T cells in peripheral blood (PB) and intestinal biopsy (IB) during eight months of cART treatment. The absolute number (**A**) and percentage (**B**) of PB CD4 T cells are shown before (M0) and after eight months (M8) of cART. Fold change over baseline (M0) of CD4 T cell absolute number (left) and percentage (right) is depicted in (**C**). Extent of intestinal CD4 T cells before (M0) and after (M8) cART (**D**). Positive correlation between the percentages of CD4 T cells in PB and IB atM0 (close circles) and M8 (open circles) (**E**).

After eight months of cART, all patients had less than 1.57 HIV-RNA log10 copies/mL in plasma. All patients showed a recovery in the levels of circulating CD4**^+^** T cells, not significant for absolute number (median cells/µl M8 vs. M0: 472 vs. 367; p = 0.13; Figure 2A) but significant for percentages of total CD4**^+^** T cells (23.0 vs. 16.0; p<0.0001; Figure 2B). When expressed as fraction variation from baseline, the achieved reconstitution of CD4**^+^** T cells was of 14,6% for absolute counts and 26% for percentages (Figure 2C). A statistically significant reconstitution was observed also for intestinal CD4^+^ T cells, which levels increased from 8.94% of total CD4**^+^** T cells at M0 to 27.47% at M8 (p = 0.0001; Figure 2D). The reconstitution of intestinal CD4**^+^** T cells was variable among the different patients, with the percentage variation from pre-cART values ranging from 5.2 to 31.0% (Figure 2D). Interestingly, a highly significant association was found between the percentages of CD4^+^ T cell in blood and those in the GI tract at baseline (filled circles; r = 0.7252, P = 0.0270, Figure 2E) and after eight months of therapy (open circles; r = 0.7349, p = 0.0241, Figure 2E), thus indicating that a common mechanism regulate the levels of CD4+ T cells in these two compartments.

### Impact of short-term cART in the frequencies of circulating and intestinal Th1 and Th17 cells

**Figure 3:**
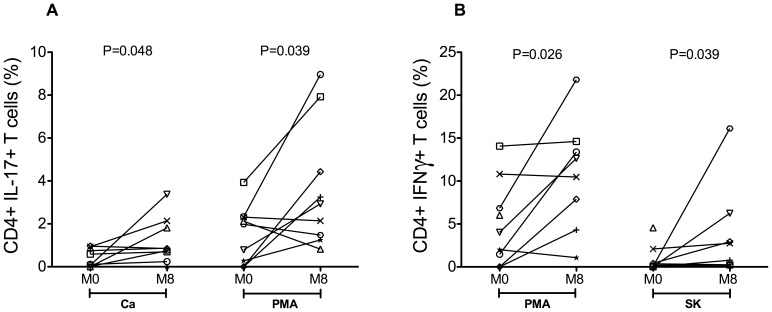
Effects of short-term cART on intestinal IL-17 or IFN-γ producing CD4 T cells. Effects of short-term cART on intestinal Th17(**A**) and Th1 (**B**) cells. Th17 and Th1 cells were identified by flow cytometry as CD4 T cells that produce IL-17(Th17) or IFN-γ (Th1) after brief in vitro stimulation with Candida albicans mannoprotein antigen (Ca), phorbolmyristyl acetate (PMA), or Streptokinase (SK).

The extent and timing of intestinal Th17 cell reconstitution in cART-treated HIV-infected individuals are not well understood. For this reason, we next sought to determine whether eight months of cART impacted on the frequencies of Th17 and Th1 cells, functionally defined as CD4**^+^** T cells producing IL-17 or IFN-γ, respectively, after *in vitro* stimulation with PMA, SK and *Ca*. In the gut, the frequencies of Th17 cells at M8 were significantly higher when compared to those observed at M0 after stimulation with *Ca* as well as PMA (p = 0.048 and p = 0.039, respectively; Figure 3A). Of note, these effects were specific for intestinal Th17 cells, since no significant differences were observed in circulating Th17 cells with any of the used stimulus (data not shown). The frequencies of intestinal Th1 cells were significantly higher at M8 when compared to M0 following stimulation with both PMA (p = 0.026) and SK (p = 0.039) (Figure 3B), but not with *Ca* (data not shown). A significant increase for Th1 cells was observed in blood only following stimulation with SK (p = 0.043), but not with PMA or *Ca* (data not shown).

**Figure 4:**
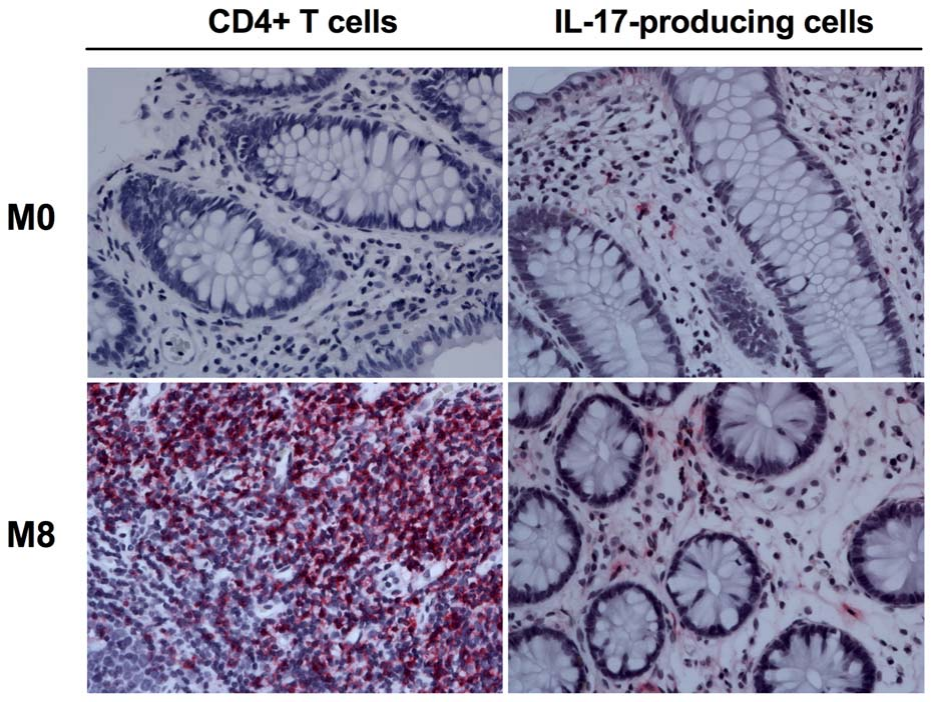
*In situ* immunohistochemistry staining for intestinal CD4T cells andIL-17 producing cells. Representative staining for CD4 (left) and IL-17 (right) in formalin-fixed paraffin-embedded colon biopsies collected before (M0, top) and after eight months (M8, bottom) of cART.

To further evaluate the effects of cART on intestinal CD4**^+^** T cells and Th17 cells, we then performed a single-label immune-histochemistry analysis on formalin-fixed paraffin-embedded colon biopsies of eight of the nine HIV positive patients before (M0) and at eight months (M8) of cART treatment. One patient was excluded because it was no longer available a sufficient amount of biological samples to perform this analysis. Representative images of intestinal cells expressing CD4 or IL-17 are showed for a representative individual in Figure 4. A precise and extensive *in situ* quantitation of CD4 or IL-17 positive cells as numbers/unit of tissue was not possible in the relatively small amount of available tissues. Thus, to better estimate the variation in the levels of CD4**^+^** T cells and IL-17-producing cells, we performed a semiquantitative analysis in which each sample was given an increasing score based on the number of positive cells analyzed in five different fields per slide (see Methods). As shown in Table 2, an increased positivity for CD4**^+^** T cells was evident in three out of eight HIV-infected individuals at M8 when compared to M0, with the remaining five individuals showing an overall stable level of CD4**^+^** T cell numbers. Interestingly, cART therapy was particularly effective in increasing the levels of IL-17-producing cells, with the positivity for IL-17 being higher at M8 than M0 in five of the eight included subjects (Table 2). Of note, the five patients with increased *in situ* levels of IL-17-producing cells also showed increased levels of IL-17 production after *in vitro* stimulation, as determined by intracellular cytokine staining. Notably, all three patients showing increased CD4**^+^** T cell levels at M8 also showed reconstitution of IL-17**^+^** cells (showed in bold in Table 2). Overall, these data indicate that the increased reconstitution of intestinal CD4**^+^** T cells achieved with eight months of cART includes Th17 and Th1 cells. Since reconstitution of intestinal Th17 cells was partial and variable between different patients, these data also suggest that a more complete reconstitution of intestinal Th17 cells would very likely require a prolonged period of treatment. Finally, these data are consistent with a model in which reconstitution of Th17 cells is a key determinant for the reconstitution of the overall intestinal CD4**^+^** T cell compartment following cART.

**Table 2:** Semiquantitative analyses of CD4+ and IL-17+ cells before (M0) and after (M8) cART.

Patient code	Experimental point	IL-17+ cells (Vector red)	CD4 cells (Vector red)
**A2**	**M0**	**+**	**+**
**A2**	**M8**	**++**	**+++**
**A6**	**M0**	**+**	**−**
**A6**	**M8**	**+++**	**+**
**A1**	**M0**	**+**	**++**
**A1**	**M8**	**++**	**+++**
A3	M0	+	++
A3	M8	++	++
A4	M0	+	++
A4	M8	++	++
A11	M0	+	++
A11	M8	+	++
A7	M0	++	+++
A7	M8	++	++
A9	M0	+++	+++
A9	M8	++	++

Slides of colon tissues were scored as “−” (less than 3 positive cells); “+” (3–10 positive cells); “++” (20–40 positive cells) and “+++” (more than 50 positive cells). **Bold:** samples in which both IL-17+ and CD4+ cells were increased at M8; Black: samples in which only IL-17+ cells were increased at M8; **Gray**: samples in which neither IL-17+ nor CD4+ cells were increased at M8.

### Impact of short-term cART in the levels of microbial translocation, T cell activation and proliferation

We next investigated the possible association between reconstitution of intestinal CD4^+^ T cells and the extent of microbial translocation, by evaluation of levels of lipopolysaccharide (LPS) and bacterial 16sDNA in plasma.LPS and bacterial 16sDNA are considered valid indicators of enterocytes damage and compromised intestinal mucosal barrier and markers of microbial translocation. Furthermore we analyzed soluble CD14 (sCD14), as marker of LPS bioactivity [41], and the fraction of T cells expressing markers of activation (CD38 and HLA-DR) and proliferation (Ki-67) at M8 and M0. Due to the limited LPL cells obtained from gut biopsies, T cell activation markers were determined only in circulating lymphocytes.

**Figure 5:**
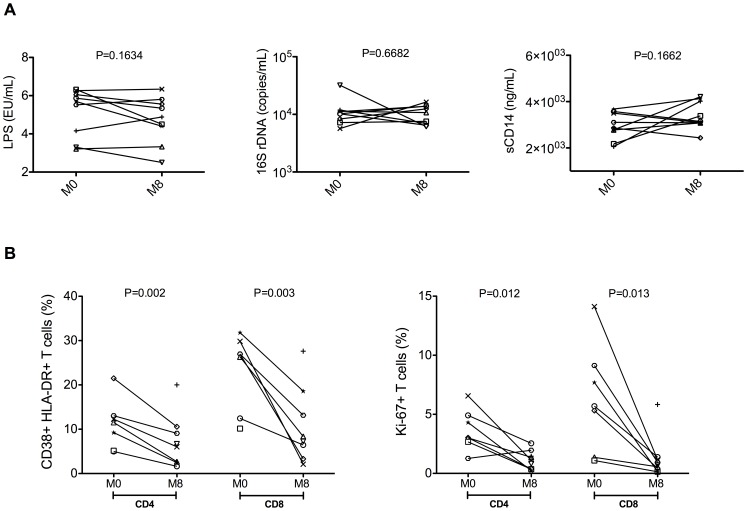
Effects of short-term cART on microbial translocation and T cell activation/proliferation. Plasma levels of lipopolysaccharide (LPS), 16S rDNA and soluble CD14 (sCD14) before (M0) and after eight months (M8) of cART (**A**). LPS and sCD14 were measured by ELISA, while 16S rDNA by PCR. Percentages of activated (as determined by expression of CD38 and HLA-DR; left), and proliferating (as determined by expression of Ki-67) staining; right) CD4 and CD8 T cells (**B**).

Eight months of cART were not able to reduce the abnormal levels of markers associated with microbial translocation and monocyte/macrophage activation, with plasma levels of LPS, 16S rDNA, or sCD14 that did not changed significantly between M0 and M8 (Figure 5A).

In contrast, cART was effective in reducing the extents of T cell activation and proliferation, assessed by expression of CD38, HLA-DR and Ki-67.

Indeed, the percentages of CD4^+^CD38^+^HLA-DR^+^ (11.1 vs 7.3; p = 0.002) and CD8^+^CD38^+^HLA-DR^+^ (23.49 vs 10.85; p = 0.003), as well as those of CD4^+^Ki-67^+^ (3.67 vs 1.12; p = 0.012) and CD8^+^Ki-67^+^ (6.34 vs 1.25; p = 0.013) T cells were significantly reduced at M8 of cART as compared to pre-treatment (Figure 5B).

### Immunological correlates of blood and intestinal CD4+ T cell reconstitution during cART

Finally, we investigated the immunological correlates of blood and intestinal CD4^+^ T cell reconstitution during cART. At M8, CD4^+^ T cell count recovery in blood was positively associated with the increase of intestinal Th17 (stimulated with SK: r = 0.857, p = 0.007) and Th1 (stimulated with SK: r = 0.857, p = 0.007; Ca: r = 0.762, p = 0.028) cells (data not shown).

In contrast, the fraction of intestinal CD4^+^ T cells were not statistically associated with the proportion of Th1 or Th17 cells neither in blood nor in GI tract (data not shown).

**Figure 6:**
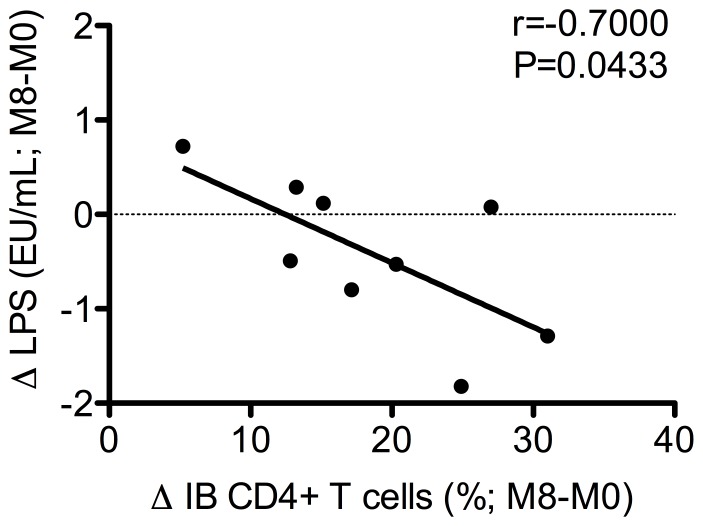
Reconstitution of intestinal CD4 T cell inversely correlates with plasma LPS level. The reconstitution of the intestinal CD4 T cells, expressed as %M8 - %M0(Δ), inversely correlates with the reduction of plasma LPS, expressed as level at M8 - level M0 (Δ).

Interestingly, we found a significant correlation between the extent of intestinal CD4^+^ T cell reconstitution (expressed as %M8 - %M0) and the reduction of plasma LPS (express as level M8 – level M0), consistent with the tight relationship between mucosal immunologic integrity and microbial translocation (r = −0.7000 p = 0.0433; Figure 6).

Of note, we did not find any significant association at M8 between the levels of intestinal Th17 or Th1 cells and those of the different markers of microbial translocation assessed (LPS, sCD14, 16S rDNA; data not shown).

## Discussion

In HIV infected individuals and SIV infected rhesus macaques, a rapid and severe depletion of intestinal CD4**+** T cells, in particular of those belonging to the Th17 subset, associates with loss of mucosal integrity, increased immune activation/inflammation, and progression to AIDS [17], [18], [24]. The importance of preserve and/or reconstitute an adequate balance of intestinal Th17 cells is highlighted by three lines of evidence. First, Th17 cells are maintained at healthy levels during non progressive HIV (long term non progressors, elite controllers) and SIV (sooty mangabeys, African green monkeys) infections [16]. Second, in rhesus macaques increased size of the Th17 compartment prior to SIV infection associates with reduced levels of SIV replication and increased mucosal integrity in the first weeks of infection [39]. Third, we recently showed that increased levels of intestinal Th17 cells induced in vivo by administration of interleukin-21 in SIV-infected rhesus macaques resulted in reduced levels of microbial translocation and systemic activation/inflammation in the chronic infection [35].

The extent to which cART is able to restore a physiological level of the intestinal CD4^+^ T cell compartment is still unclear [12], [40]–[42]. The paucity of data is particularly evident for the functional subsets of CD4^+^ T cells such as Th17 and Th1.

In trying to provide useful information to address this important question, we have conducted a longitudinal study in which the levels of total, Th17 and Th1 CD4^+^ T cells were determined in peripheral blood and pooled biopsies – obtained from terminal ileum, right colon, left colon and sigmoid colon – from nine HIV-infected individuals before and at eight months post cART initiation. Strengths of our study are: i) the large sampling of the colon performed in HIV-infected patients at two different time points – i.e. prior and at eight months of cART; ii) the assessment of intestinal Th17 and Th1 cell subsets and iii) the possibility to quantify IL-17 producing cells directly *in situ* as well as *in vitro* using different stimulations. Due to the longitudinal nature of the study, we considered the treatment “effective” if the fraction of intestinal CD4^+^ T cells or Th17 cells were significantly higher at eight months of cART as compared to the pre-cART value.

We observed a complete virological response and a significant increase in the absolute count and fraction of blood CD4^+^ T cells in all treated patients. Intestinal CD4^+^ T cells were also significantly higher at M8 when compared to M0, although the extent of reconstitution was variable between the different individuals, ranging from modest (+5,21%) to substantial (+31%).

Interestingly, we observed a significant association between the percentages of CD4^+^ T cells in blood and GI tract at M8 of cART. These data are consistent with a previous report in which CD4^+^ T cells were compared in blood and GI tract of HIV-infected individuals, and suggest a common mechanism regulating the homeostasis of CD4^+^ T cells in these two compartments during HIV infection [26].

The frequency of CD4**+** cells producing IL-17 following stimulation with PMA was very low in most of our patients, both in blood and in GI tract. This is not surprising, since Th17 cells are preferentially lost in HIV infection, and their depletion occur in the early stage of infection [12], [25], [43]. The frequencies of blood Th17 cells did not significantly increase in cART-treated individuals with any of the tested stimulus. However, the levels of intestinal Th17 cells at M8 were significantly higher than those at M0 following stimulation with *Ca* and, although to a less extent, with PMA. The effective reconstitution of intestinal CD4^+^ T cells and Th17 cells was confirmed directly *in situ* by immune-histochemistry staining for CD4 and IL-17 in the colon of eight of the nine HIV-infected individuals that completed the study. Semi-quantitative image analyses at M0 and M8 showed a substantial cART-induced recovery of CD4^+^ T cells and of Th17 cells in three and five of these eight patients, respectively. Of note, all three patients showing increased *in situ* levels of CD4**+** T cell also showed significant reconstitution of IL-17 producing cells at M8. These data are consistent with a model, proposed by Macal and colleagues, in which reconstitution of Th17 cells is critical for the reconstitution of the overall intestinal CD4^+^ T cell compartment following cART.

Importantly, there was high consistency between the IHC and the flow cytometry results, with the five patients showing increased *in situ* staining for IL-17- producing cells also showing increased fraction of CD4^+^ T cells expressing IL-17 after *in vitro* stimulation [44].

The recovery of IFN-γ expressing cells was more consistent, with significant increased levels of IFNγ-producing CD4**+** T cells in response to SK stimulus in both peripheral and intestinal districts.

These observations suggest that Th1 cells can be restored earlier than Th17 cells during cART, and are consistent with the data from Mavigner and colleagues suggesting an impairment of Th17 cell homing to the GI tract of cART-treated HIV-1 infected subjects [13].

The recovery of intestinal CD4**+** T cells during cART has been previously studied, with controversial results and/or interpretation of the results. Earlier longitudinal and cross-sectional studies concluded that cART, even when initiated during primary infection, was poorly effective in reconstituting intestinal CD4**+** T cells [45]–[48].

In contrast, more recent studies have showed the efficacy of cART to induce an effective CD4^+^ T cell reconstitution in gut mucosa [26], [43], [49].

Several factors complicate these studies and their interpretation. First, due to the difficulty to perform two or more mucosal collections from the same subjects, the large majority of studies was cross-sectional and did not allow a direct comparison between pre-cART and post-cART inside the same individuals. Second, due to the difficulty to perform multiple invasive sampling in colon, most of the observations come from studies in which only recto sigmoid or jejunal region of the intestine were sampled. Third due to the absence of a control group; however, it is important to note that the parameters determined in this study – level of CD4^+^ T cells, Th17 cells, immune activation - are usually stable in untreated, chronically HIV infected patients in the short time window of this study, or change in the opposite direction – CD4^+^ T cells and Th17 cells decrease and immune activation increase – compared to those described in this study with introduction of ART. Fourth due to the laboratory techniques, in fact the semiquantitative analysis of the IHC staining for CD4^+^ T cells and IL-17^+^ cells, combined with the limited size of the intestinal biopsies, may have limited our ability to detect additional, less pronounced differences between pre-ART and post-ART samples. Finally, there is no agreement on the definition of “effective” reconstitution.

This problem is particular important for cross-sectional studies, in which the levels of CD4**^+^** T cells or Th17 cells in cART-treated HIV-infected individuals need to be compared with those found in untreated HIV-infected individuals and/or healthy controls. While numerous reports showed reduced frequencies of intestinal Th17 cells in HIV-infected individuals with active viral replication [12], [25], [28], [29], the efficacy of cART in reconstituting intestinal Th17 cells are somehow less clear, with studies showing inadequate reconstitution of Th17 cells in long-term cART treated HIV infected individuals when compared with healthy controls as well as a substantial restoration of intestinal Th17 cells in a subgroup of individuals with more than five years of cART and high levels of mucosal CD4^+^ T cells [27], [44]. In a recent cross-sectional study, Chege and colleagues showed an overall normalization of sigmoid Th17 cells on people treated with cART for more than four 18 years [12]. However, there was a considerable inter-individual variability in Th17 reconstitution, with higher frequencies of sigmoid Th17 cells being associated with reduced microbial translocation [12]. Thus, loss of Th17 cells might persist in the GI tracts despite long-term cART and it may critically contribute to the persistence of microbial translocation.

It is important to note that the HIV-infected individuals included in our study started cART with a relatively short exposure to the virus - median time of 2 years from HIV infection diagnosis - and a relatively high median of CD4^+^ T cell count (367 cells/ml). These characteristics may have been critical for the good immunologic response achieved with only eight months of cART, and these findings support the concept that early treatment improves immune reconstitution in HIV infected patients [50]. Indeed, a recent study showed that patients who started cART before six months after infection had lower levels of T-cell activation (as determined by fraction of T cells expressing CD38^+^ and HLA-DR^+^) and smaller reservoir size (as determined by cell associated HIV-DNA and HIV-RNA levels) compared to individuals who began treatment after more than two years of infection [9]. A limitation of our study is the lack of virological data on mucosal tissues. Considering that at mucosal sites low levels of viral replication can still be observed after years of cART initiation, it is reasonably to hypothesize that eight months of therapy could have only partially suppressed active viral replication in the GI tract of our patients [47], [51], [52].

We also investigated how eight months of cART impacted on the levels of immune activation/inflammation, as well as the direct relationship between the repopulation of intestinal CD4^+^ T cells and the extent of residual immune activation/inflammation. In our study, short-term cART significantly decreased the levels of CD4^+^ and CD8^+^ T cell activation, as measured by expression of HLA-DR and CD38, and proliferation, as measured by expression of Ki-67, in the blood of HIV-infected individuals. Unfortunately, the size of the colon biopsies that we were able to collect precludes us to determine the expression of these activation/proliferation markers at mucosal site. Although effective in reducing systemic T cell activation and proliferation, eight months of virological suppressive cART was not sufficient to significantly reduce plasma levels of LPS, sCD14 or bacterial 16sRNA.

Furthermore, we found a significant association between the recovery of CD4 cells in gut and the lowering of LPS levels in blood, consistent with a tight biological link between restoration of mucosal immune functions and reduction of microbial translocation to the systemic circulation. Elevated LPS levels has been correlated with monocyte activation, and may contribute to chronic immune activation during HIV infection [10]. These data are consistent with previous studies showing that the levels of microbial translocation decrease during cART but very rarely normalize even after a prolonged treatment, indicating a probably impaired intestinal mucosal repair and regeneration in HIV infection [10], [28], [53]–[60]. In our study with did not find significant changes in sCD14 levels, marker of monocyte response to LPS, but, as already observed by others [10], it is possible that few months of ARV could not be effective in lowering sCD14 plasma concentration. Finally, we found that the magnitude of intestinal Th17 cell reconstitution at M8 associates directly with blood CD4^+^ T-cell counts.

This finding comes with two important limitations. First, we did not determine the levels of intestinal T cell activation/proliferation, thus we cannot exclude a local effect in which increased levels of CD4^+^ T cell or Th17 cell reconstitution correlate with reduce immune activation at mucosal site. Second, the short-term cART (eight months) and the relatively small number of subjects analyzed could have limited our power to detect statistically significant correlation. Moreover, we have not follow up data after eight months of antiviral therapy at this moment, anyway we planned a further time of follow up at two years of treatment to verify the hypothesis that a more complete constitution of intestinal Th17 cells would very likely require a prolonged period of treatment.

In conclusion, this longitudinal study in which different areas of the colon were sampled in HIV infected patients prior and at eight months of cART showed a significant recovery of intestinal CD4^+^ T and a partial restoration of the critical Th17 cell subsets during short-term cART.

Importantly, this study confirmed the biological link between mucosal immune function and microbial translocation, and propose pre-cART levels of blood CD4**+** T cell counts and activated CD8^+^ T cells as critical determinant of Th17 cell reconstitution at mucosal sites. Thus, these findings expend the important impact of cART on mucosal immunity of HIV-infected patients, and provide further rationale for an early initiation of cART.

## Supporting Information

Checklist S1 **Checklist of the study.**
(DOCX)

Protocol S1 **Protocol of the study in English.**
(DOC)

Protocol S2 **Protocol of the study in Italian (original language).**
(DOC)
