# Supplementary figures and images for: Reconstitution of Intestinal CD4 and Th17 T Cells in Antiretroviral Therapy Suppressed HIV-Infected Subjects: Implication for Residual Immune Activation from the Results of a Clinical Trial
Source: PLoS One. 2014 Oct 23;9(10):e109791. doi: 10.1371/journal.pone.0109791 (PMC4207675; doi:10.1371/journal.pone.0109791)

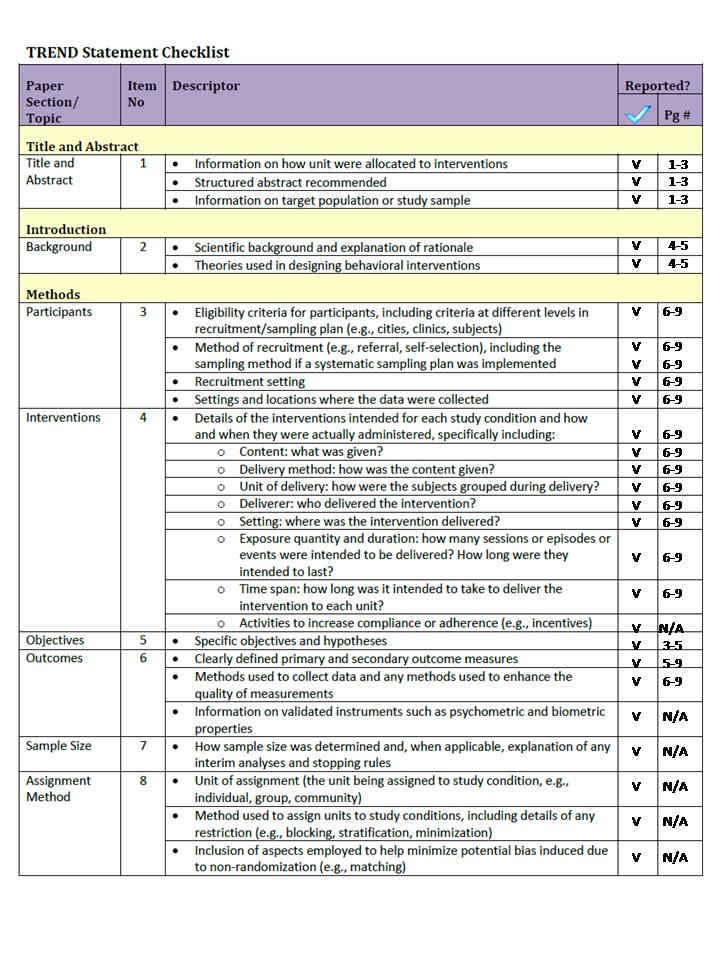


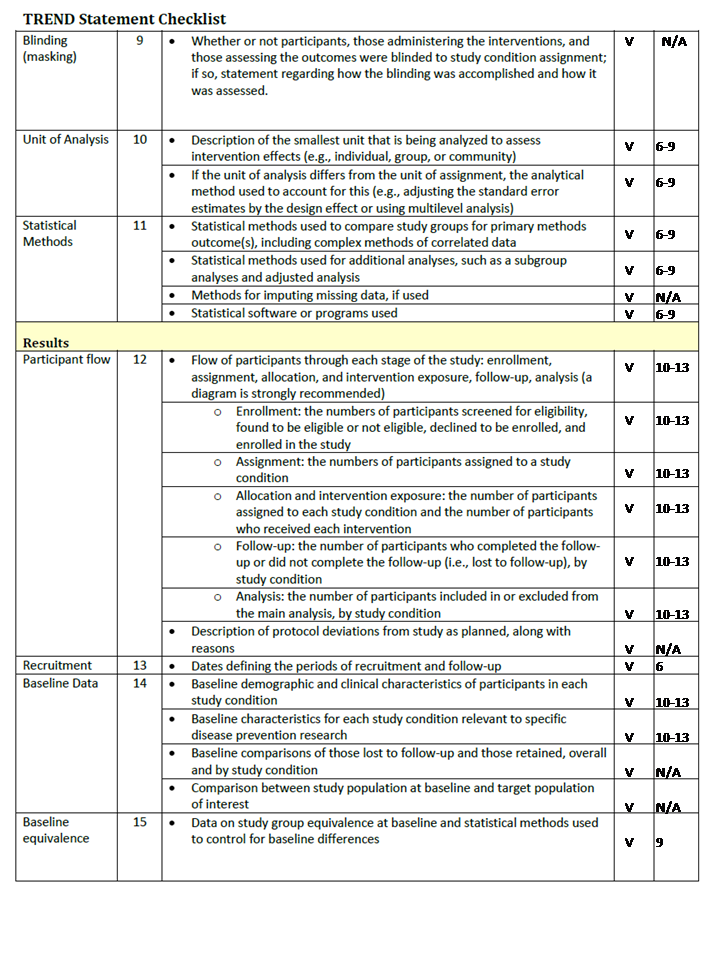


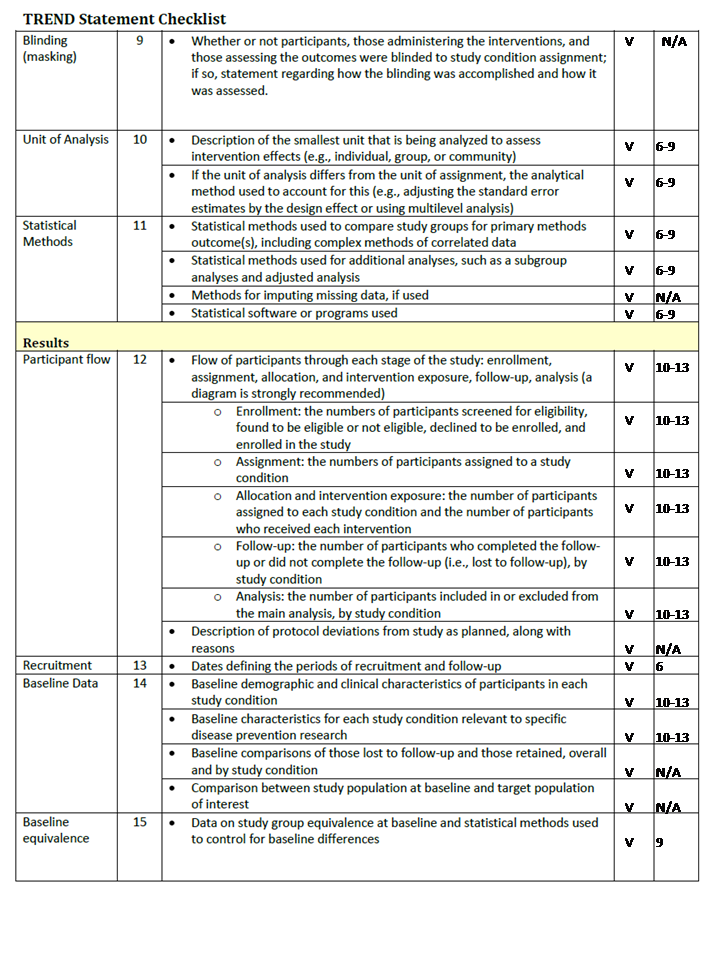

Supplement: Checklist S1 — Checklist of the study. (DOCX) [file pone.0109791.s001.docx]
