# Supplementary material for: Reconstitution of Intestinal CD4 and Th17 T Cells in Antiretroviral Therapy Suppressed HIV-Infected Subjects: Implication for Residual Immune Activation from the Results of a Clinical Trial
Source: PLoS One. 2014 Oct 23;9(10):e109791. doi: 10.1371/journal.pone.0109791 (PMC4207675; doi:10.1371/journal.pone.0109791)
Supplement: Protocol S1 — Protocol of the study in English. (DOC) [file pone.0109791.s002.doc]

**Study of the intestinal mucosa immunity in HIV infection**

PRINCIPAL INVESTIGATOR: PROFESSOR VINCENZO VULLO

PROTOCOL DATE: 22-02-2009

Version: 1.1

**INTRODUCTION AND OBJECTIVES**

**1 . Background:**

The global HIV/AIDS pandemic is one of the greatest tragedies of modern times with over 40 million infected individuals worldwide and between 3 and 4 million deaths each year. However, despite 20 years of research by the international scientific community, the pathogenic mechanisms of HIV infection are still poorly understood. The observations that: a) HIV infects and kills CD4+ T-cells , b) the progressive depletion of CD4+ T-cells following HIV infection , c ) the level of viral replication predicts the rate of depletion of T cells CD4+ , has led many scientists to conclude that AIDS is the result of direct HIV- mediated killing of CD4+ T-cells (1). Other important findings, however, have questioned this model of pathogenesis. In particular it was found that in HIV positive patients the rate of cells which undergo death is increased both for subpopulations CD4 + and CD8+, and that the majority of T cells - CD4+ that die during HIV infection are not infected by the virus. This increase in necrosis of T cells not infected seems, however, be correlated with high levels of chronic activation of T cells that occur in the course of HIV infection. Therefore it has been proposed that the HIV-related chronic immune activation plays a key role in the pathogenesis of AIDS (2-5). The observation that the levels of immune activation are able to better predict the survival compared to the levels of viral replication in HIV positive patients, supports this hypothesis in a substantial way (6).

Important contributions to the understanding of the pathogenesis of HIV/AIDS have come from studies on infection by non-pathogenic SIV in natural hosts such as Sooty Mangabeys (SMs) (7). In stark contrast to infection by pathogenic lentivirus , SIV infection of natural Mangabeyes is not associated with a disease similar to AIDS despite many years of infection and high levels of viral replication. The experimental infection with SIV of Asian monkeys Rhesus Macaques (different from natural host) evolves with the appearance of symptoms similar to those described in patients with AIDS (simian AIDS). While the mechanisms underlying non-pathogenic SIV infection in the natural host are still unclear, it is becoming increasingly clear that the understanding of these mechanisms could lead to considerable steps forward in explaining the pathogenesis of AIDS in humans. In previous studies it has been highlighted that a key step of not pathogenic SIV infection in SMs is the absence of chronic, generalized immune activation that is associated with disease progression in HIV-infected patients (8-9). The results of these studies on infection by non-pathogenic SIV in SMs provide strong theoretical support to the hypothesis that, even in the pathogenesis of AIDS in humans, the immune activation is a key determinant of disease progression. However it should be pointed out that the differences in the mechanisms involved in the immune activation in humans and SMs remain poorly understood . The CD4+ memory T-cells are a heterogeneous population of lymphocytes that play an essential role in the functioning of the immune system. Have been identified 3 main groups of memory CD4 + T-cells ( a) T-helper cells that produce IFN -1 – gamma, TNF and IL-2 ; ( b ) T-helper -2 cells that produce IL -4 , IL- 5 and IL-13 ; ( c ) T regulatory cells that produce TGF-beta and IL-10 . More recently, a new CD4 + cell subset was identified on the basis of the preferential production of pro -inflammatory cytokine called IL-17 ( 10-11). The subset of CD4+ T-lymphocytes that produce IL-17 is distinct from Th1 , Th2 , and T regulatory ; its differentiation from naive precursors is specifically induced by IL -6 and TGF- beta (12). The CD4 + T helper cells that produce IL-17 were shown to be able to promote a pro-inflammatory "immune environment", in particular at the mucosal level where they also seem to regulate the function of the epithelial barrier of the mucosa (13-14).

Many recent studies have shown that infections by pathogenic HIV/SIV are associated with early and severe depletion of CD4+ T-cells in the mucosal tissue (15-20). For these reasons, it is proposed a model in which this depletion of CD4+ T-cells in the mucosal level may play a role in the pathogenesis of AIDS (15-20).

In agreement with this model, the acute HIV infection is characterized by a massive and irreversible depletion of CD4+ memory T cells-CCR5+ at the level of the mucosa. This depletion of mucosal CD4+ T-cells and the consequent loss of T-helper cells may lead to the overcoming of the intestinal mucosal barrier by enteric bacteria: this may result in an increase in the patient's susceptibility to subclinical gastrointestinal infections. The ineffectiveness of the mucosal immune system thus favors the establishment of chronic HIV-related immune activation and eventually can develop into systemic collapse of the immune system which is associated with AIDS.

More recently, attention has been focused on the effects of immunological non-pathogenic SIV infection in SMs in the mucosa associated lymphoid tissue, MALT (20). It was carried out a detailed analysis of T cells derived from rectal biopsies and broncho alveolar washes that have shown that the SMs infected with SIV show a significant depletion of mucosal CD4 T-cells. The SMs infected with SIV, in an interesting way, show levels of mucosal and systemic immune activation similar to that of non-infected animals. Therefore, it was concluded that natural not pathogenic SIV infection in SMs is it characterized by a depletion of CD4 T-cells in the MALT; nevertheless this infection remain asymptomatic because of the absence of the phenomena of chronic immune activation.

**2 . Objectives:**

These studies are the background of the present project that will explore, in HIV positive patient , the relationship between the depletion of mucosal CD4+ T-cells , the mucosal and systemic immune activation and progression of HIV disease.

The objective is to analyze the hypothesis that in the events leading to the onset of AIDS in humans, the role of mucosal and / or systemic immune activation is as important as the role of depletion of mucosal CD4+ T-cell mediated by the virus.

In addition to this primary objective, the current project aims to study the role of Th-17 cells. The rationale is based on the hypothesis that differences in the levels and function of circulating Th-17 cells are involved in determining the differences in the levels of immune activation that distinguish pathogenic infection in humans from the non-pathogenic in SMs. As a corollary, this study explore if the modifications of mucosal levels of Th17 cells may contribute to immune activation and / or to the loss of mucosal integrity in the context of HIV infection. The rationale for this hypothesis comes from the observation that Th-17 cells have a potentially pro inflammatory phenotype that makes them attractive candidates for mediating systemic and mucosal HIV related immune activation. A small number of data on the role of Th-17 cells during HIV infection are described in the literature, therefore, we believe that the experiments proposed in the protocol will be an important source of information on the mechanisms responsible for chronic generalized immune activation that is associated with HIV infection.

Finally, the promoters of this study believe and hope that the results of the project will be useful in designing new and better therapeutic strategies for the treatment of HIV infection.

Primary objective:

1 ) Carry out an analysis aimed at understanding the changes in the levels of activation of T cells in the mucosal and systemic HIV-positive patients at an earlier stage at the start of antiretroviral therapy ( naive ) and then compared with the beginning of therapy (approximately 6 months).

Secondary objectives:

Investigate on :

1 ) the presence of correlations between the levels of systemic and mucosal immune activation,

2 ) the extent of lymphocyte depletion of CD4+ T cells at mucosal level,

3 ) the role of IL- 17 produced by CD4+ T cells through a longitudinal analysis of the levels of IL-17 in peripheral blood and intestinal mucosa of HIV+ subjects in pre-and post - therapy

4) the potential correlation between IL-17 produced by CD4 + T cells and the main markers of progression of HIV disease , including the immunological response to antiretroviral therapy (HAART).

**3 . Participating centers :**

Patients will be enrolled in the Department of Infectious and Tropical Diseases , University " Sapienza" of Rome under the supervision of Prof Vincenzo Vullo ( Professor of Infectious Diseases and Director of the Department ) . The blood samples will be carried out by the nursing staff at the Day Hospital of Infectious Diseases.

The endoscopy will be performed at the Department of Surgery " Pietro Valdoni " of the Policlinico Umberto I in Rome ( Prof Fiocca ) .

Biological samples collected will be studied in the laboratories of the Department of Infectious and Tropical Diseases , University " Sapienza" of Prof. Vincenzo Vullo and at the Stellar -Chance Laboratories , University of Pennsylvania - USA ( Prof Guido Silvestri ) .

**STUDY DESIGN**

4 . Study design and methodology :

Longitudinal pilot study . Experimental medications or experimental diagnostic procedures will be not used in this study.

5 . Duration:

The project will have a total duration of 24 months: approximately 6-12 months are required for enrollment. From the follow-up of the last patient in the study, completion of laboratory tests is expected in approximately 6 months.

**PATIENT SELECTION**

**6 . Criteria for inclusion and exclusion :**

Inclusion criteria:

1 ) Patients aged between 18 and 60 years

2 ) Patients who have signed the informed consent

3 ) HIV-positive patients naïve to antiretroviral therapy

Exclusion criteria:

1 ) Treatment with systemic corticosteroids and / or inhaled corticosteroids or use of immunomodulatory drugs including interferon and hydroxyurea.

2 ) current or previous neoplastic disease

3 ) History of inflammatory bowel disease (including ulcerative colitis, celiac disease and Whipple's disease) and other non-infectious diseases of the gastrointestinal tract .

4 ) Diarrhea (defined as two or more watery stools per day) for 14 days prior to enrollment

5 ) anemia , pregnancy or any other contraindication to blood sampling

6 ) Use of anticoagulants

Each study participant may withdraw consent to participation at any time.

**7 . Vulnerable population :**

Will not be included in the study population vulnerable .

**8 . Patient recruitment and screening :**

10 HIV-infected patients naive to antiretroviral therapy, that achieve the clinical indication to start antiretroviral therapy, will be enrolled. For enrollment no procedures for randomization are required. The recruitment will be carried out gradually: we will enroll the first 10 patients who meet the criteria for inclusion and exclusion criteria for this study and who agree to participate in the trial by signing the informed consent.

**9 . Data and biological samples :**

**a.) Data:**

The laboratory analysis that will be made will include (but not be limited to ): 1 ) flow cytometric analysis of activation, proliferation and differentiation markers of lymphocytes; 2 ) measurement of plasma levels of cytokines ; 3 ) morphological studies ( histological , immunohistochemical and in situ hybridization) ; 4) virological studies .

**b. ) Biological samples :**

Biological samples collected during the study will be limited to peripheral blood and intestinal biopsies obtained by colonoscopy. The endoscopist will get intestinal biopsies during each colonoscopy (three in the terminal ileum , three from the right colon , three from the left colon , three from the sigma) .

No genetic testing will be carried out in the course of this study. For this protocol there is no provision of: 1) storage and preservation of the tissues collected for a longer period of time allotted for the study ; 2) generation of cell lines. No clinical data and / or biological sample will be identified with the identity of the patient in question.

**PROCEDURES**

**10 . procedures :**

The participation in this protocol requires only the following: 1 ) blood samples , 2) Two colonoscopies for each patient 3 ) medical checkups . Each procedure provided in this study will be performed only after the patient has signed the informed consent.

**Blood Sampling**

The blood samples (30-40 ml of venous blood at each monitoring) will be carried out at the Department of Infectious Diseases by the nursing staff.

**Colonoscopy**

The patient will be subjected to two endoscopic procedures in approximately six months (T0 and T1). During colonoscopy, patients will be adequately sedated.

Intestinal biopsies will be taken during each colonoscopy (three in the terminal ileum , three from the right colon , three from the left colon , three from the sigma). The rationale for making withdrawals in four different areas of the intestine (terminal ileum, right colon, left colon, sigmoid colon) is based on evidence of heterogeneity in the different portions of the intestinal mucosa of the gastrointestinal tract . Based on recent studies indicating that the mucosal immune system is the key to the anatomical site of disease progression from HIV / SIV ( Mehandru et al. , J Exp Med 2004; Brenchley et al. , J Exp Med 2004; Mattapallil et al . , Nature 2005; Haase et al. , Nature 2005; Guadalupe et al. , J Virol . , 2006) is currently of great scientific importance of the continuation of studies on the effects of HIV mucosal immunity. The rationale for the withdrawal of three intestinal biopsies for each area lies in the fact that , based on our experience as well as that of our colleagues, a biopsy is needed to carry out the studies required by flow cytometry ( which will allow to characterize the immunophenotype of immune cells ) and two biopsies will be used to perform morphological studies / histology (which require a sample of paraformaldehyde fixation and paraffin embedding and the other sample freezing ). Biological samples will be collected for FACS analysis , for histological and virological analysis. The endoscopic procedure will be performed under sedation and requires about 30 minutes. At the end of the endoscopic examination, in the absence of clinical contraindications and after adequate medical observation, the patient will be discharged.

**Flow Cytometry**

The samples for analysis by FACS will be processed following standard procedures : biopsy will be placed in the culture medium supplemented with fetal bovine serum and antibiotics. The tissue will be destroyed mechanically and passed through a filter before the resuspension of the cells obtained . The mucosal cells will be separated from the epithelial cells by Percoll density gradient . An analysis will be carried out by flow cytometry with FACScan .

histological studies

Include basic morphological studies , the in situ hybridization (ISH ) and immunochemistry (IHC) . For each patient, two biopsies collected will be processed and fixed for histological studies which approximately include: 1 ) morphological analysis , 2 ) search for the replication of the virus by ISH , 3) IHC for the detection of T-cell CD4 + and their activation rate or proliferation with simultaneous staining of HLA -DR , or Ki67 , quantification of apoptotic cells using the TUNEL assay in situ .

STATISTICAL ANALYSIS

11 . Determination of sample size and statistical methods :

The determination of sample size is not possible in this pilot study because they are not available in the literature data regarding the primary objective of the study population. 10 HIV-positive patients will be studied before the start of antiretroviral therapy (naive) and approximately after 6 months of antiretroviral therapy . A descriptive analysis will be performed on the data collected.

SAFETY AND ADVERSE EVENTS

12 . Potential Hazards:

The risks for the collection of peripheral blood include discomfort , bleeding or hematoma formation in the site of the body where the needle is introduced, and a small risk of infection. Currently there are no known additional risks.

In general colonoscopy is a safe procedure but are present some risks associated with the procedure and with the use of sedation . The risks associated with the procedure ranging from a minimal to clinically significant problems . Patients may notice bleeding and cramping after the procedure . Significant risks include: 1) intestinal bleeding (5 cases per 1000 procedures ), 2) intestinal perforation (3 cases per 1000 procedures ) . If the patient has significant bleeding may require one or more transfusions ; in the case of bowel perforation may require hospitalization , the administration of antibiotics and / or the ' surgery . The risks associated with anesthesia include 1 ) local reactions at the site of insertion of the venous catheter ( phlebitis ), 2) allergic reactions , 3) relatively rare complications of cardiac and respiratory function . The overall risk is low and is less than 1 in every 100 patients.

13 . Potential benefits :

This study does not provide direct benefits for people enrolled. However, the persons employed will be able to :

• benefit in the future of scientific information obtained in the course of this study.

• derive personal satisfaction from participating in studies that contribute to the understanding the immune system and to search for new methods to prevent and treat HIV / AIDS.

• be familiar with the clinical course of their infection and the conditions of their immunity by analysis that are not routinely available in the management of HIV positive patient .

14 . Patient Protection :

This protocol is not based on the use of experimental drugs or experimental devices .

The amount of blood drawn is considerably below the amount that can be considered dangerous for the health of the patient. In addition, patients with anemia , pregnancy or with any other clinical contraindication for withdrawal will not be considered eligible for this study. Finally, the sampling procedure will be performed in its entirety by professional staff trained and in a secure environment . Even the endoscopic procedure , in order to minimize the risk of complications , will be carried out by medical personnel, with proven experience and in a protected environment . All those who have an added risk for the endoscopic procedure will also be excluded from the study: those that use 1 ) systemic or inhaled corticosteroids , 2 ) immunomodulatory , including interferon and hydroxyurea, 3 ) those who have submitted or will submit neoplasms , 4) people with inflammatory bowel disease ( including ulcerative colitis, celiac disease and Whipple's disease ) and other non-infectious diseases of the gastrointestinal tract , 5) diarrhea (defined as two or more watery stools per day) for 14 days prior to enrollment .

This Protocol provides for the protection of the patient through insurance company .

15 . adverse Events

In case of occurrence of adverse events during the procedures, all medical supports needed to solve the problem will be implemented. Moreover all those who present contraindications to the performance of these diagnostic procedures will not be subjected to these tests. All adverse events will be recorded in accordance with applicable laws .

The program includes reporting to the coordinating center and the respective Ethics Committee of any severe adverse event (SAE ) defined by the presence of at least one of the following conditions :

• fatal

• life-threatening

• severe or permanent disability

• requires hospitalization or prolongation of hospitalization in progress

Such reporting shall be made no later than 24 hours with regard to SAE with fatal outcome or injurious life threatening. For all other SAE is possible to make the notification within 7 days from the presentation of the event.

16 . Confidentiality and collection of clinical data and biological samples :

All information related to participation in this program will be treated as strictly confidential in accordance with the standards of good clinical practice ( Decree of the Ministry of Health April 99 and supplements) , as well as for the protection of persons and other subjects regarding the processing of personal Data ( Legislative Decree no. 196 /03).

The data of each patient , collected during the study will be recorded on paper forms (CRF) and on a computer database. The documents collected will be stored in a protected space in the Department of Infectious and Tropical Diseases , University " Sapienza" of Rome. The computer database of clinical data created to perform the statistical analysis will be protected by adequate computer security tools. It will be ensured at all times patient privacy and access to data will be protected by the Investigator . The data store will be available for inspection by the regulatory authorities (Ministry of Health ), and the authorities in charge of the verification of the correct procedures ( Independent Ethics Committee ) without , however, there is the possibility to trace the identity of the patients. So also for biological samples will be guaranteed absolute anonymity and will be free from any element useful in discovering the identity of the patient. The materials will be kept for the time necessary to carry out studies in the laboratories of the Department of Infectious and Tropical Diseases , University La Sapienza - Policlinico Umberto I in Rome , following all safety regulations prescribed by law and by the rules of "good -practice " . The sending of biological materials at the Stellar -Chance Laboratories , University of Pennsylvania - USA ( Prof Guido Silvestri ) will take place following all national and international regulations provided for such services and will respect both the criteria of both biological safety regulations regarding privacy of the patient ( will not be transmitted sensitive data) .

17 . risk / benefit ratio :

This study represents a lot more from a scientific and medical point of view with respect to the minimal risk associated with the proposed endoscopic procedure . The risk of participation in the study is balanced by the high potential benefit that the results of the study will give the participants themselves and the whole general population.

ETHICAL ASPECTS AND INFORMED CONSENT

18 . Evaluation of the study complies with the ethical principles

The protocol will be submitted to the approval of the Independent Ethics Committee of the Policlinico Umberto I in Rome responsible for the evaluation of the projects of the Site Coordinator . All information related to participation in this study will be treated with strict confidentiality in accordance with the standards of good clinical practice ( Decree of the Ministry of Health April 99 and supplements) , as well as for the protection of persons and other subjects regarding the processing of personal Data ( Legislative Decree no. 196 /03).

19 . Procedures to request informed consent :

Written informed consent will be required to participate in the study each patient : endoscopic procedures , blood samples and the acquisition of the patient's clinical data provided in the study will be made only after signing the informed consent. Participation in the study is subject to the understanding by the patient of the content and purpose of the study and signing the informed consent . Informed consent will be signed at the Department of Infectious and Tropical Diseases of the University of Rome " La Sapienza in the presence of one of the investigators participating in the study ." A necessary condition to give informed consent is the literacy of the patient and his ability to understand.

FEES , CONFLICTS OF INTEREST AND LOANS

20 . Compensation for subjects enrolled in the study :

There are no economic compensation for people who agree to be enrolled in the study .

21 . Conflicts of interest

All the people involved in the study have no conflicts of interest of a financial nature.

22 . Financing of the study

Clinical study spontaneous , non-profit .

SCIENTIFIC PUBLICATIONS

23 . Scientific publications :

The results of the study will be analyzed , submitted for publication in scientific journals and presented at international meetings and conferences.

REFERENCES

1 . Stevenson M. Nat . Med 2003; 9:853-60 .

2 . McCune JM. Nature 2001; 410:974-979 .

3 . Z. Grossman et al. , Nat Med 2002; 8:319-323 .

4 . G. Silvestri et al . , J Clin Invest 2003; 112:821-824 .

5 . Moanna A. et al. , Curr HIV / AIDS Report , 2005; 2:16-23 .

6 . Giorgi J.V. et al . , J Infect Dis , 1999. 179:859-70 .

7 . Silvestri G. Primatol J Med , 2005 , 34:243-252

8 . G. Silvestri et al. , Immunity 2003; 18 : 441-452 .

9 . G. Silvestri et al . J Virol 2005; 79:4043-54 .

10 . Harrington L.E. et al. , Nat Immunol 2005; 6: 1123-1132 .

11 . H. Park et al. , Nat Immunol 2005; 6: 1133-1141 .

12 . M. Veldhoen et al. , Immunity 2006; 24: 179-189 .

13 . S. Schwartz et al. , Biochem & Biophys Res Commun 2005; 337 : 505-509 .

14 . S. Fujino et al. , Gut 2003; 52 (1) :65 -70.

15 . Brenchley J. et al. , J Exp Med 2004; 200:749-59 .

16 . Mehandru S. et al. , J Exp Med 2004; 200:761-70 .

17 . Picker L.J. et al . Nat Immunol 2005; 6:430-2 .

18 . Haase HV et al. , Nat Rev Immunol 2005; 5 : 783-792 .

19 . Brenchley J.M. et al. , Nat Immunol 2006; 7 ( 3 ) : 235-239 .

20 . Z. Grossman et al. , Nat Med 2006; 12 (3): 289-295 .
