# Supplementary material for: Reconstitution of Intestinal CD4 and Th17 T Cells in Antiretroviral Therapy Suppressed HIV-Infected Subjects: Implication for Residual Immune Activation from the Results of a Clinical Trial
Source: PLoS One. 2014 Oct 23;9(10):e109791. doi: 10.1371/journal.pone.0109791 (PMC4207675; doi:10.1371/journal.pone.0109791)
Supplement: Protocol S2 — Protocol of the study in Italian (original language). (DOC) [file pone.0109791.s003.doc]

**PROTOCOLLO TRIAL CLINICO**

**Studio dell’immunita’ mucosale nell’infezione da HIV**

**INTRODUZIONE E OBIETTIVI**

1. Background:

La pandemia di AIDS è una delle più grandi tragedie dei tempi moderni con oltre 40 milioni di individui infettati nel mondo e tra i 3 e i 4 milioni di morti ogni anno. Tuttavia, nonostante 20 anni di ricerche da parte della comunità scientifica internazionale, i meccanismi patogenetici dell’infezione da HIV restano ancora scarsamente compresi.

Le osservazioni che: a) l’HIV infetta e uccide le cellule T-CD4+, b) la deplezione progressiva delle cellule T-CD4+ segue l’infezione da HIV, c) il livello di replicazione virale predice il tasso di deplezione delle cellule T-CD4+, ha portato molti scienziati a concludere che l’AIDS è il risultato del killing diretto HIV-mediato delle cellule T-CD4+ (1). Altre importanti scoperte tuttavia hanno messo in discussione questo modello patogenetico. In particolare è emerso che nei pazienti HIV positivi il tasso di cellule che vanno incontro a morte è incrementato sia per le sottopopolazioni CD4+ che per le CD8+, e che la maggior parte delle cellule T-CD4+ che muoiono durante l’infezione da HIV sono non infettate dal virus. Questa incremento della necrosi delle cellule T non infettate sembra, invece, essere correlata con gli alti livelli di attivazione cronica delle cellule T che si verificano nel corso dell’infezione da HIV. Pertanto è stato proposto che l’immunoattivazione cronica HIV-relata giochi un ruolo chiave nella patogenesi dell’AIDS (2-5). Supporta in modo sostanziale questa ipotesi l’osservazione che, nei pazienti HIV positivi, i livelli di immunoattivazione sono in grado di predire meglio la sopravvivenza rispetto ai livelli di replicazione virale (6).

Importanti contributi alla comprensione della patogenesi dell’AIDS sono venuti da studi sull’infezione non patogenica da SIV in ospiti naturali come le Sooty Mangabeys (SMs) (7). In netto contrasto con le infezioni patogeniche da lentivirus, l’infezione naturale da SIV dei Mangabeyes non è associata con una malattia simile all’AIDS nonostante lunghi anni di infezione e alti livelli di replicazione virale. L’infezione sperimentale con SIV di ospiti non naturali come le specie di scimmie asiatiche (Rhesus Macaques) si evolve con la comparsa di sintomi simili a quelli descritti nei pazienti con AIDS (simian AIDS). Mentre i meccanismi alla base dell’infezione non patogenetica di SIV nell’ospite naturale restano ancora poco chiari, appare sempre più evidente che la comprensione di tali meccanismi potrebbe consentire notevoli passi avanti nella spiegazione della patogenesi dell’AIDS nell’uomo. In precedenti studi è stato evidenziato come un passaggio chiave dell’infezione non patogenica da SIV nelle SMs sia proprio l’assenza della immunoattivazione cronica e generalizzata che è associata con la progressione della malattia nei soggetti HIV positivi (8-9). I risultati di questi studi sull’infezione non patogenica da SIV nelle SMs forniscono un forte supporto teorico all’ipotesi che, anche nella patogenesi dell’AIDS nell’uomo, l’immunoattivazione sia un determinante chiave della progressione della malattia. Tuttavia va sottolineato come le differenze nei meccanismi implicati nell’immunoattivazione nell’uomo e nelle SMs restano scarsamente compresi. Le cellule T-CD4+ memory sono una popolazione eterogenea di linfociti che giocano un ruolo essenziale nel funzionamento del sistema immunitario. Sono stati individuati tre set principali di cellule T-CD4+ memory (a) cellule T-helper-1 che producono IFN-γ, TNF e IL-2; (b) cellule T-helper-2 che producono IL-4, IL-5 e IL-13; (c) cellule T-regolatorie che producono TGF-β e IL-10. Più recentemente un quarto subset cellulare di CD4+ è stato identificato sulla base della produzione preferenziale di una citochina pro infiammatoria denominata IL-17 (10-11). Il subset di linfociti T-CD4+ che produce IL-17 è distinto dalle cellule Th1, Th2, e T-regolatorie; la sua differenziazione dai precursori naive è specificamente indotta da IL-6 e TGF-β (12).

Le cellule T helper CD4+ che producono IL-17 hanno mostrato di saper promuovere un “ambiente immune” pro infiammatorio, in particolare a livello mucosale dove sembrano anche regolare la funzione della barriera epiteliale della mucosa (13-14).

Molti recenti studi hanno mostrato che le infezioni patogeniche da HIV/SIV sono associate ad una precoce e severa deplezione delle cellule T-CD4+ nel tessuto mucosale (15-20).

Per tali ragioni è stato proposto un modello nel quale questa deplezione di cellule T-CD4+ a livello mucosale possa ricoprire un ruolo determinante nella patogenesi dell’AIDS (15-20).

In accordo con questo modello l’infezione acuta da HIV è caratterizzata da una massiva e irreversibile deplezione delle cellule T-memory CD4+CCR5+ a livello della mucosa.

Questa deplezione mucosale di cellule T-CD4+ e la conseguente perdita di cellule T-helper può determinare il superamento della barriera mucosale intestinale da parte dei batteri enterici: ciò può tradursi in un aumento della suscettibilità del paziente a infezioni gastrointestinali subcliniche. L’inefficacia del sistema immune mucosale favorisce quindi lo stabilirsi di un’immunoattivazione cronica HIV-relata ed eventualmente può evolvere nel collasso sistemico del sistema immunitario che è associato con l’AIDS conclamato.

Più recentemente è stata focalizzata l’attenzione sugli effetti immunologici dell’infezione non patogenica di SIV nelle SMs nei tessuti linfoidi associati alla mucosa, MALT (20). È stata effettuata un’analisi dettagliata delle cellule T ricavate da biopsie rettali e da lavaggi bronco alveolari che hanno evidenziato come le SMs infettate da SIV mostrano una significativa deplezione delle cellule T-CD4 mucosali. Le SMs infettate da SIV, in modo interessante, mostrano tuttavia livelli di immunoattivazione mucosale e sistemica simile a quello di animali non infetti. Pertanto si è giunti alla conclusione che l’infezione naturale non patogenica di SIV nelle SMs è si caratterizzata da una deplezione delle cellule T-CD4 nel MALT, ma che tuttavia rimane asintomatica a causa della assenza di fenomeni di immunoattivazione cronica.

1. Obiettivi

Questi studi hanno rappresentato pertanto il background dell’attuale progetto di esplorare, nel paziente HIV positivo, la relazione tra la deplezione mucosale delle cellule T-CD4+, l’immunoattivazione mucosale e sistemica e la progressione della malattia da HIV.

L’obiettivo è analizzare l’ipotesi che negli eventi che portano al manifestarsi dell’AIDS nell’uomo, il ruolo dell’immunoattivazione mucosale e/o sistemica sia importante tanto quanto il ruolo delle deplezione mucosale delle cellule T-CD4+ virus-mediata.

In aggiunta a questo obiettivo primario, l’attuale progetto si propone di studiare il ruolo delle cellule Th-17. Il razionale si basa sul ipotesi che le differenze nei livelli e nella funzione delle cellule Th-17 circolanti siano coinvolti nel determinare le differenze nei livelli di immunoattivazione che distinguono l’infezione patogenica nell’uomo da quella non patogenica nelle SMs. A corollario il presente studio ipotizza che le modificazioni livello mucosale dei livelli di cellule Th17 possano contribuire all’immunoattivazione e/o alla perdita dell’integrità mucosale nel contesto dell’infezione da HIV. Il razionale per tale ipotesi nasce dall’osservazione che le cellule Th17 hanno un differente fenotipo potenzialmente pro infiammatorio che le rende un interessante candidato per mediare l’immunoattivazione HIV relata sia a livello sistemico che mucosale. Sono descritti in letteratura un esiguo numero di dati sul ruolo delle cellule Th17 nel corso di infezione da HIV pertanto riteniamo che gli esperimenti proposti nel protocollo saranno un importante fonte di informazione sui meccanismi responsabili della immunoattivazione cronica generalizzata che si associa con un’infezione da HIV.

Infine i promotori di tale studio ritengono e si augurano che i risultati del progetto siano utili nel disegnare nuove e migliori strategie terapeutiche per il trattamento dell’infezione da HIV.

Obiettivo primario:

1) Effettuare una analisi indirizzata a comprendere le modificazioni dei livelli di attivazione delle cellule T mucosali e sistemiche nei pazienti HIV positivi in una fase precedente all'inizio della terapia antiretrovirale (naive) e successivamente rispetto all'inizio della terapia (6 mesi).

Obiettivi secondari:

Investigare nei pazienti HIV su:

1) la presenza di correlazioni tra i livelli di immunoattivazione sistemici e mucosali,
2) l'entità della deplezione linfocitaria delle cellule T CD4+ a livello mucosale,
3) Investigare sul ruolo dell’IL-17 prodotta dalle cellule T CD4+ attraverso un’analisi longitudinale dei livelli di IL-17 prodotti dalle cellule T CD4+ nel sangue periferico e nella mucosa intestinale dei soggetti HIV+ in fase pre e post-terapia ( circa 6 mesi),

4) investigare sulla potenziale correlazione tra IL-17 prodotta dalle cellule T CD4+ e i principali markers di progressione della malattia da HIV, inclusa la risposta immunologica alla terapia antiretrovirale (HAART).

1. Centri partecipanti:

I pazienti saranno arruolati presso il Dipartimento di Malattie Infettive e Tropicali dell’Università “Sapienza” di Roma sotto la supervisione del Prof Vincenzo Vullo (Professore Ordinario di Malattie Infettive e Direttore del Dipartimento). I prelievi ematici programmati verranno effettuati dal personale infermieristico in servizio presso il centro prelievi del Day Hospital di Malattie Infettive.

Le endoscopie saranno effettuate presso il Dipartimento di Chirurgia “Pietro Valdoni” del Policlinico Umberto I di Roma (Prof Fiocca).

I campioni biologici raccolti saranno studiati presso i laboratori del Dipartimento di Malattie Infettive e Tropicali dell’Università “Sapienza” di Roma del Prof. Vincenzo Vullo e presso gli Stellar-Chance Laboratories dell’University of Pennsylvania - USA (Prof Guido Silvestri).

**DISEGNO DELLO STUDIO**

1. Disegno dello studio e metodologia:

Studio pilota longitudinale. Non verranno utilizzati in tale studio farmaci o procedure clinico diagnostiche sperimentali.

1. Durata:

Il progetto avrà la durata complessiva di 24 mesi: Dall’ultimo follow-up dell’ultimo paziente in studio sono previsti 6 mesi per il completamento degli esami di laboratorio.

**SELEZIONE DEI PAZIENTI**

1. Criteri di inclusione e di esclusione:

**Criteri di inclusione:**

1) Pazienti di età compresa tra18-60 anni

2) Pazienti che abbiano firmato il consenso informato

3) Pazienti HIV positivi naive per terapia antiretrovirale

**Criteri di esclusione:**

1) Trattamento con corticosteroidi sistemici e/o inalatori oppure uso di farmaci immunomodulatori inclusi interferone e idrossiurea.

2) Malattia neoplastica attuale o pregressa

3) Storia di malattia infiammatoria intestinale (incluse colite ulcerosa, morbo celiaco e malattia di Whipple) e altre patologie non infettive del tratto gastrointestinale.

4) Diarrea (definita come due o più scariche di feci liquide al giorno) nei 14 giorni precedenti all’arruolamento

5) Anemia, gravidanza o qualunque altra controindicazione al prelievo ematico

6) Uso di anticoagulanti

Ciascun partecipante allo studio può ritirare in qualunque momento il proprio consenso alla partecipazione alla sperimentazione.

1. Popolazione vulnerabile:

Non saranno inclusi nella popolazione arruolata soggetti vulnerabili.

1. Arruolamento dei pazienti e screening:

Verranno arruolati 10 individui HIV positivi naive per terapia antiretrovirale e che presentino l’indicazione clinica a iniziare la terapia antiretrovirale. Per l’arruolamento non sono previste procedure di randomizzazione. L’arruolamento sarà effettuato progressivamente; verranno arruolati i primi 10 pazienti che risponderanno ai criteri di inclusione ed esclusione previsti per questo studio e che accetteranno di partecipare alla sperimentazione firmando il consenso informato.

1. Dati e campioni biologici:
   1. Dati:

Le analisi laboratoristiche che verranno effettuate includeranno (ma non saranno limitate): 1) all’analisi in citometria a flusso dei markers di attivazione, proliferazione e differenziazione linfocitaria; 2) alla misura dei livelli plasmatici di citochine; 3) allo studio morfologico (istologico, immunoistochimico e con ibridazione in situ); 4) agli studi virologici.

- 1. Campioni biologici:

I campioni biologici raccolti nel corso dello studio saranno limitati al sangue periferico e alle biopsie intestinali ottenute mediante colonscopia. L’endoscopista otterrà biopsie intestinali nel corso di ciascuna colonscopia (tre a livello dell’ileo terminale, tre dal colon destro, tre dal colon sinistro, tre dal sigma).

Non saranno effettuati test genetici nel corso di questo studio.

Non sono previsti per questo protocollo: 1) stoccaggio e conservazione dei tessuti raccolti per un periodo maggiore del tempo previsto per lo studio; 2) generazione di linee cellulari.

Nessun dato clinico e/o campione biologico sarà identificabile con l’identità del paziente in esame.

**PROCEDURE**

1. Procedure:

La partecipazione a questo protocollo prevede esclusivamente: 1) prelievi ematici, 2) due colonscopie per ciascun paziente 3) visite mediche di controllo. Ciascuna procedura prevista in questo studio verrà effettuata solo dopo che il paziente abbia firmato il consenso informato.

***Prelievo di sangue***

Verranno prelevati a ciascun controllo previsto 30-40 ml di sangue venoso. I prelievi ematici verranno effettuati presso il Dipartimento di Malattie Infettive a cura del personale infermieristico.

***Colonoscopia***

Il paziente sarà sottoposto a due procedure endoscopiche a circa sei mesi di distanza l’una dall’altra. Nel corso della colonscopia i pazienti saranno adeguatamente sedati.

Saranno prelevate biopsie intestinali nel corso di ciascuna colonscopia (tre a livello dell’ileo terminale, tre dal colon destro, tre dal colon sinistro, tre dal sigma). Il razionale per effettuare prelievi in quattro differenti aree dell’intestino (ileo terminale, colon destro, colon sinistro, sigma) è basato sull’evidenza dell’eterogeneità della mucosa intestinale nelle differenti porzioni del tratto gastrointestinale. Sulla base di recenti studi che indicano che il sistema immune mucosale è il sito anatomico chiave per la progressione della malattia da HIV/SIV (Mehandru et al., J Exp Med 2004; Brenchley et al., J Exp Med 2004; Mattapallil et al., Nature 2005; Haase et al., Nature 2005; Guadalupe et al., J Virol. 2006), è attualmente di straordinaria importanza scientifica la prosecuzione degli studi sugli effetti dell’HIV sull’immunità mucosale. Il razionale per il prelievo di tre biopsie per ciascuna area intestinale risiede nel fatto che, sulla base della nostra esperienza così come su quella dei nostri colleghi, una biopsia è necessaria per effettuare gli studi in citofluorimetria previsti (che consentiranno di caratterizzare l’immunofenotipo delle cellule immunitarie) mentre due biopsie serviranno per svolgere gli studi morfologici/istologici (che richiedono per un campione la fissazione in paraformaldeide e l’inclusione in paraffina e per l’altro campione il congelamento). I campioni biologici saranno raccolti per l’analisi al FACS, per gli esami istologici e virologici. La procedura endoscopica sarà effettuata in sedazione e richiederà circa 30 minuti per essere completata. Al termine dell’esame endoscopico, in assenza di controindicazioni cliniche e dopo adeguata osservazione medica il paziente verrà dimesso.

***Citometria a flusso***

I campioni destinati all’analisi con il FACS saranno processati seguendo le procedure standard: il materiale bioptico verrà posto in medium di coltura supplementato con siero bovino fetale e antibiotici. Il tessuto verrà distrutto meccanicamente e passato attraverso un filtro prima della raccolta e della risospensione delle cellule ricavate. I linfociti mucosali verranno separati dalle cellule epiteliali mediante gradiente di densità con Percoll. Sarà effettuata un analisi in citometria a flusso con FACscan.

***Studi istologici***

Includono gli studi morfologici di base, l’ibridizazione in situ (ISH) e l’immunochimica (IHC). Per ciascun paziente due delle biopsie raccolte saranno processate e fissate per gli studi istologici che includono: 1) analisi morfologica, 2) ricerca della replicazione del virus mediante ISH, 3) IHC per la ricerca delle cellule T-CD4+ e del loro tasso di attivazione o proliferazione con simultaneo staining dell’HLA-DR o Ki67, quantificazione delle cellule apoptotiche usando il TUNEL assay in situ.

**ANALISI STATISTICA**

1. Determinazione della dimensione del campione e metodi statistici:

La determinazione della dimensione del campione non è possibile in questo studio pilota in quanto non sono disponibili in letteratura dati di popolazione riguardo all’obiettivo primario dello studio.

Verranno pertanto studiati 10 pazienti HIV positivi in una fase precedente all'inizio della terapia antiretrovirale (naive) e a circa 6 mesi rispetto all'inizio della terapia stessa. Sui dati raccolti, verrà effettuata l’analisi descrittiva.

**SICUREZZA ED EVENTI AVVERSI**

1. Rischi potenziali:

I rischi per il prelievo di sangue periferico includono fastidio, sanguinamento o possibile formazione di ematoma nel sito del corpo dove viene introdotto l’ago per il prelievo e un piccolo rischio di infezione. Attualmente non sono noti ulteriori rischi.

In generale la colonscopia è un procedimento sicuro ma sono tuttavia presenti alcuni rischi associati con la procedura e con l’uso della sedazione. I rischi associati con la procedura vanno da un minimo fastidio a problemi clinicamente significativi. I pazienti possono notare dopo la procedura sanguinamenti e crampi. I rischi significativi includono: 1) sanguinamento intestinale (5 casi ogni 1000 procedure), 2) perforazione intestinale (3 casi ogni 1000 procedure). Se il paziente presenta un sanguinamento significativo può essere necessaria una o più trasfusioni; nel caso di perforazione intestinale può essere necessaria l’ospedalizzazione, la somministrazione di antibiotici e/o l’ intervento di chirurgia. I rischi connessi con l’anestesia includono 1) reazioni locali a livello del sito di inserzione del catetere venoso (flebiti), 2) reazioni allergiche, 3) complicazioni relativamente rare della funzione cardiaca e respiratoria. Il rischio complessivo è basso ed è di meno di 1 ogni 100 pazienti.

1. Potenziali benefici:

Questo studio non prevede effetti benefici diretti per le persone arruolate.

Tuttavia grazie alla partecipazione a questo studio le persone arruolate potranno:

- giovarsi in futuro delle informazioni scientifiche ottenute nel corso di questo studio.
- trarre soddisfazione personale dall’aver partecipato a studi che contribuiscono alla comprensione del funzionamento del sistema immunitario e alla ricerca di nuovo metodi per prevenire e trattare un’importante malattia infettiva come l’HIV/AIDS.
- conoscere l’andamento clinico della loro infezione e le condizioni della loro immunità mediante analisi che non sono disponibili di routine nella gestione del paziente HIV positivo.

1. Tutela del paziente:

Questo protocollo non prevede l’uso di farmaci o device sperimentali.

La quantità di sangue prelevata è considerevolmente al di sotto della quantità che può essere ritenuta pericolosa per la salute del paziente. Inoltre i pazienti con anemia, in gravidanza o con ogni altra controindicazione clinica per il prelievo non saranno considerati elegibili per questo studio. Infine la procedura di prelievo verrà effettuata in ogni sua parte da personale professionale adeguatamente formato e in un ambiente protetto. Anche la procedura endoscopica, al fine di ridurre al minimo il rischio di complicanze, verrà svolta da personale medico professionale adeguatamente formato, con comprovata esperienza e in ambiente protetto. Inoltre verranno esclusi dallo studio tutti coloro che presentano un rischio aggiunto per la procedura endoscopica: coloro che usino 1) corticosteroidi sistemici o inalatori, 2) immunomodulanti, inclusi interferone e idrossiurea, 3) coloro che abbiano presentato o presentino neoplasie, 4) persone con malattie infiammatorie intestinali (incluse colite ulcerosa, morbo celiaco e malattia di Whipple) e altre patologie non infettive del tratto gastrointestinale, 5) diarrea (definita come due o più scariche di feci liquide al giorno) nei 14 giorni precedenti all’arruolamento.

Il presente protocollo prevede la tutela del paziente mediante copertura assicurativa aziendale.

1. Eventi avversi

In caso di comparsa di eventi avversi nel corso delle procedure diagnostiche previste nello studio saranno messi in atto tutti presidi medico-chirurgici necessari per risolvere il problema. Per altro non verranno sottoposti a tali esami tutti coloro che presentino controindicazioni all’esecuzione di tali procedure diagnostiche. Tutti gli eventi avversi saranno registrati in conformità alle leggi vigenti.

Il programma prevede la segnalazione al centro coordinatore e al rispettivo Comitato Etico di ogni evento avverso severo (SAE) definito dalla presenza di almeno una delle seguenti condizioni:

• esito fatale

• pericolo di vita

• disabilità severa o permanente

• necessità di ospedalizzazione o prolungamento del ricovero in corso

Tale segnalazione dovrà avvenire entro e non oltre le 24 ore per quanto riguarda le SAE con esito fatale o arrecanti pericolo di vita. Per tutte le altre SAE è possibile effettuare la comunicazione entro 7 giorni dalla presentazione dall’evento.

1. Riservatezza e raccolta dei dati clinici e dei campioni biologici:

Tutte le informazioni connesse alla partecipazione al presente programma saranno trattate in modo strettamente riservato in conformità alle norme di buona pratica clinica (Decreto Ministero della Sanità aprile 99 ed integrazioni), nonché a quelle per la tutela delle persone e di altri soggetti rispetto al trattamento dei dati personali (D.Lgs. 196/03).

I dati di ciascun paziente, rilevati nel corso dello studio, saranno registrati su schede cartacee (CRF) e su un database informatizzato. I documenti raccolti saranno archiviati all’interno di uno spazio protetto nel Dipartimento di Malattie Infettive e Tropicali dell’Università “Sapienza” di Roma. Il database informatico dei dati clinici creato per effettuare l’elaborazione statistica sarà protetto da adeguati strumenti di sicurezza informatica e comunque privo di qualsiasi elemento utile a risalire all’identità del paziente. Sarà garantita in ogni momento la privacy del paziente e l’accesso ai dati sarà protetto dallo Sperimentatore. L’archivio dati sarà disponibile all’ispezione sia delle autorità regolatorie (Ministero della Sanità), sia delle autorità addette alla verifica delle corrette procedure (Comitato Etico Indipendente) senza che tuttavia vi sia la possibilità di risalire alla identità personale dei pazienti. Ugualmente anche per i campioni biologici sarà garantito l’assoluto anonimato e saranno privi di qualsiasi elemento utile a risalire all’identità del paziente. I materiali saranno conservati per il tempo necessario ad effettuare gli studi presso i laboratori del Dipartimento di Malattie Infettive e Tropicali dell’Università Sapienza– Policlinico Umberto I di Roma seguendo tutte le normative di sicurezza previste dalla legge e dalle norme di “good-practice”. L’invio dei materiali biologici presso gli Stellar-Chance Laboratories dell’University of Pennsylvania - USA (Prof Guido Silvestri) avverranno seguendo tutte le normative nazionali e internazionali previste per tali trasporti e rispetteranno sia i criteri di sicurezza biologica sia le normative relative alla privacy del paziente (non verranno trasmessi dati sensibili).

1. rapporto rischio/beneficio:

Questo studio rappresenta molto di più da un punto di vista scientifico e medico rispetto al minimo rischio associate con la procedura endoscopica proposta. Il rischio della partecipazione allo studio è bilanciato dall’elevato beneficio potenziale che i risultati dello studio potranno dare ai partecipanti stessi e a tutta la popolazione generale.

**ASPETTI ETICI E CONSENSO INFORMATO**

1. Valutazione della conformità dello studio ai principi etici

Il protocollo sarà sottomesso all’approvazione del Comitato Etico Indipendente dell’Azienda Policlinico Umberto I di Roma competente per la valutazione dei progetti del Sito Coordinatore. Tutte le informazioni connesse alla partecipazione al presente studio saranno trattate in modo strettamente riservato in conformità alle norme di buona pratica clinica (Decreto Ministero della Sanità aprile 99 ed integrazioni), nonché a quelle per la tutela delle persone e di altri soggetti rispetto al trattamento dei dati personali (D.Lgs. 196/03).

1. Procedure di richiesta consenso informato :

Il consenso informato scritto sarà richiesto a ciascun paziente che parteciperà allo studio: le procedure endoscopiche, i prelievi ematici e l’acquisizione dei dati clinici del paziente previsti nello studio verranno effettuati solo dopo la firma del consenso informato. La partecipazione allo studio è subordinata alla comprensione da parte del paziente dei contenuti e delle finalità dello studio e alla firma del consenso informato. Il consenso informato verrà firmato presso il Dipartimento di Malattie Infettive e Tropicali del Università di Roma “Sapienza alla presenza di uno degli sperimentatori partecipanti allo studio”. Condizione necessaria per dare il consenso informato è l’alfabetizzazione del paziente e la sua capacità di intendere e volere.

**COMPENSI, CONFLITTI DI INTERESSE E FINANZIAMENTI**

1. Compensi per i soggetti arruolati nello studio:

Non sono previsti compensi di natura economica per le persone che accettano di essere arruolate nello studio.

1. Conflitti di interesse

Tutte le persone coinvolte nello studio non hanno conflitti di interesse di carattere economico.

1. Finanziamento dello studio

Studio clinico spontaneo, no profit.

**PUBBLICAZIONI SCIENTIFICHE**

1. Pubblicazioni scientifiche:

I risultati dello studio verranno analizzati, sottoposti per la pubblicazione a riviste scientifiche internazionali e presentati a meeting e conferenze.

**BIBLIOGRAFIA**

1. Stevenson M. Nat. Med. 2003; 9:853-60.

2. McCune JM. Nature 2001; 410:974-979.

3. Grossman Z. et al., Nat Med 2002; 8:319-323.

4. Silvestri G. et al., J Clin Invest 2003; 112:821-824.

5. Moanna A. et al., Curr HIV/AIDS Report 2005; 2:16-23.

6. Giorgi J.V. et al., J Infect Dis, 1999. 179:859-70.

7. Silvestri G. J Med Primatol, 2005, 34:243-252

8. Silvestri G. et al., Immunity 2003; 18: 441-452.

9. Silvestri G. et al. J Virol 2005; 79:4043-54.

10. Harrington L.E. et al., Nat Immunol 2005; 6: 1123-1132.

11. Park H. et al., Nat Immunol 2005; 6: 1133-1141.

12. Veldhoen M. et al., Immunity 2006; 24: 179-189.

13. Schwartz S. et al., Biochem & Biophys Res Commun 2005; 337: 505-509.

14. Fujino S. et al., Gut 2003; 52(1):65-70.

15. Brenchley J. et al., J Exp Med 2004; 200:749-59.

16. Mehandru S. et al., J Exp Med 2004; 200:761-70.

17. Picker L.J. et al. Nat Immunol 2005; 6:430-2.

18. Haase A.T. et al., Nat Rev Immunol 2005; 5: 783-792.

19. Brenchley J.M. et al., Nat Immunol 2006; 7(3): 235-239.

20. Grossman Z. et al., Nat Med 2006; 12(3): 289-295.
